# Identification of Bacterial Taxa Present in a Concrete Pennsylvania Bridge

**DOI:** 10.1128/mra.00211-23

**Published:** 2023-05-08

**Authors:** Patricia Tadley, Alexa Bennett, Kirstin Hansen, Thomas Hanson, Joseph Colosi, Lara K. Goudsouzian

**Affiliations:** a Department of Biology, DeSales University, Center Valley, Pennsylvania, USA; b School of Marine Science and Policy, University of Delaware, Newark, Delaware, USA; University of Maryland School of Medicine

## Abstract

Concrete contains low microbial biomass, but some bacteria can grow in this highly alkaline environment. We used silica-based DNA extraction and 16S rRNA sequence analysis to identify the bacteria in a corroded concrete bridge sample from Bethlehem, Pennsylvania. Staphylococcus, Streptococcus, *Corynebacterium*, *Leifsonia*, *Vicinamibacterales*, and *Actinophytocola* were the most abundant genera.

## ANNOUNCEMENT

Concrete is a common building material used to construct most bridges in the United States. The deterioration of it over time results in bridge failure (1). The structural integrity of concrete bridges is challenging to ascertain (2). Concrete is alkaline, anoxic, and subject to environmental extremes, but some microorganisms survive inside this material (3, 4). The composition of these bacterial communities remains largely unknown. By characterizing the microbiome of a corroded bridge fragment, we hope to advance the use of concrete-dwelling bacteria as biomarkers of structural integrity.

We obtained a concrete fragment from the Minsi Trail Bridge in Bethlehem, Pennsylvania (40.6176°N, 75.3586°W). After soaking it in 10% bleach for 10 min to decontaminate the surface, we extracted DNA per reference 5 from 10 g of pulverized, decalcified concrete. Freed DNA was bound to silica, washed, and eluted in 10 mM Tris. We analyzed an interior piece of concrete, a surface piece of concrete, and a process control of sterilized glass beads.

16S rRNA amplicons (V4 region) were produced by a two-step method (6). The first PCR used primers 515F (5′-GTGYCAGCMGCCGCGGTAA-3′) (7) and 806R (5′-GGACTACNVGGGTWTCTAAT-3′) (8) modified to contain a head sequence (5′-GCTATGCGCGAGCTGC-3′) (6). The second PCR barcoding primer paired to the head sequence. Each reaction contained 1.0 μM forward and reverse primers in SuperFi II master mix with denaturation at 95°C for 30 s, annealing at 60°C for 30 s, and extension at 72°C for 60 s for a total of 30 or 5 cycles for first PCR or second PCR, respectively. Equimolar samples were purified with sparQ PureMag beads (QuantaBio). The library was prepared with the Nextflex rapid DNA sequencing (DNA-Seq) kit 2.0 (Perkin Elmer) and sequenced with MiSeq reagent kit v3, 2 × 300 bp (Illumina) (University of Delaware DNA Sequencing and Genotyping Center). Sequences were demultiplexed per reference 6 (Python 3.10.10, QIIME2 2021.2), and amplicon sequence variants (ASVs) were resolved per DADA2 pipeline tutorial v1.16 (filter and trim through remove chimeras and assign taxonomy; DADA2 v1.21.0) with trim position determined via FASTX-Toolkit v0.0.14 FASTQ-Statistics as median Q30 of all samples (9–11). Taxonomy was assigned using the RDP classifier and Silva 138 SSU Ref NR 99 database (12, 13). Default parameters were used unless otherwise specified. Due to the low biomass of concrete, the risk of contamination from laboratory microorganisms is high (14). Sample ASV observations were retained if greater than 10 times the control abundance.

**FIG 1:**
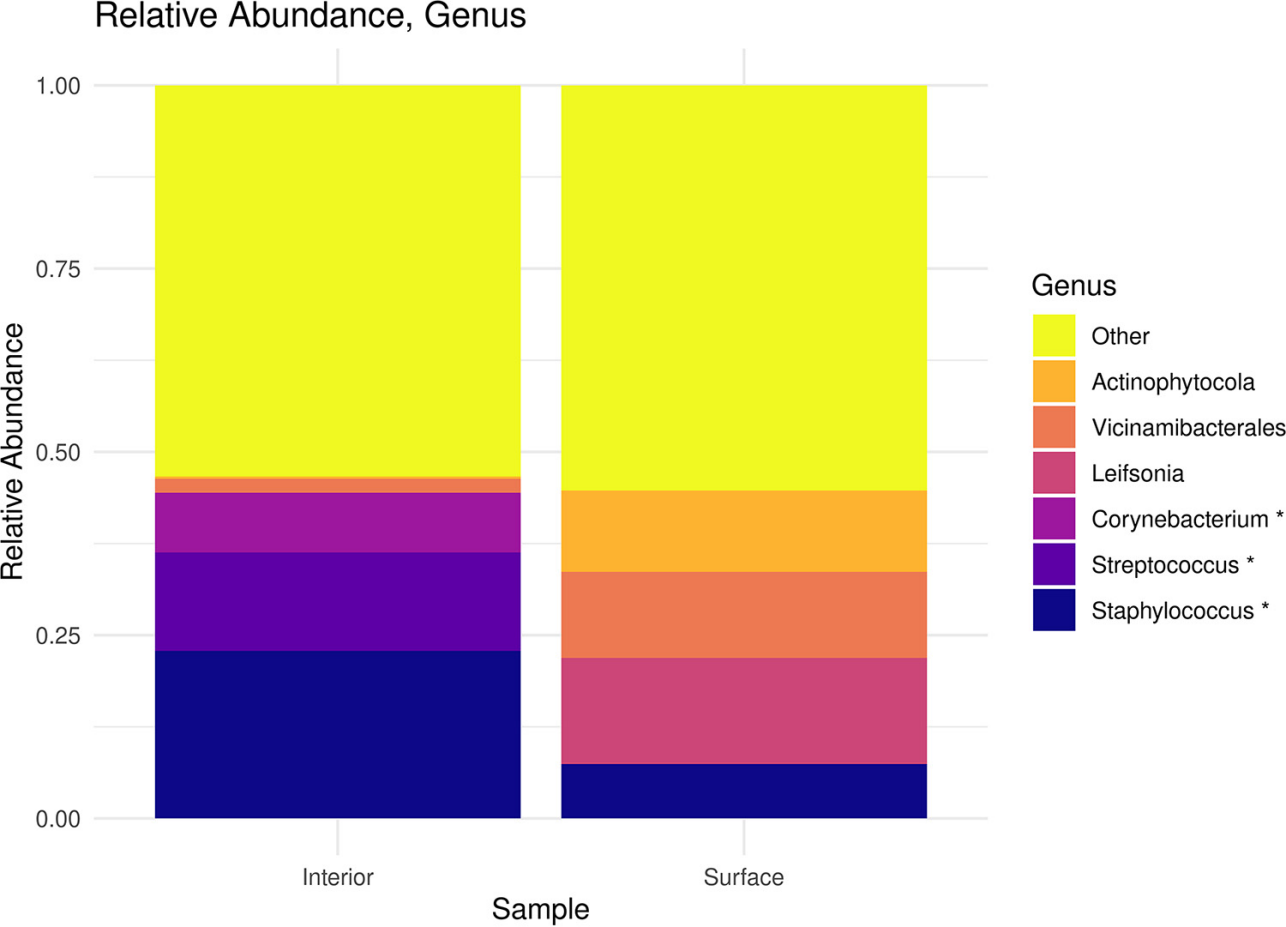
Histogram of within-sample relative abundance at the genus level after contaminant removal. The *x* axis represents the sample location relative to the concrete fragment, and the *y* axis represents within-sample relative abundance as proportions. Named genera represent the six most abundant members from the interior and surface samples. *, Genera associated previously with bacterial communities of cement powder (4).

The sequencing of all samples yielded 3,047 observations within 221 ASVs and then was reduced to 1,278 observations within 82 ASVs after contaminant removal. Only 2 of the ASVs were observed in both samples. However, these ASVs represented 23.2% and 18.5% of the interior and surface sample observations, respectively (Table 1). The most abundant genera in the interior sample were Staphylococcus, Streptococcus, and *Corynebacterium*, which were demonstrated previously to be the primary bacterial community constituents in cement powder (4). The most abundant genera in the surface sample were *Leifsonia*, *Vicinamibacterales*, and *Actinophytocola*, which were not previously associated with bacterial communities in concrete (Fig. 1) (3, 4).

**TABLE 1:** Within-sample summaries^*a*^

Sample	No. of observations prefiltration	No. of observations postfiltration	No. of ASVs postfiltration	Common ASV: Staphylococcus (%)	Common ASV: *Actinophytocola* (%)	Other ASVs (%)
Interior	1,055	954	56	22.9	0.3	76.8
Surface	464	324	28	7.4	11.1	81.5
Control	1,528					
Total	3,047	1,278	82^*b*^			

a No. of ASVs postfiltration indicates the number of ASVs after filtration remaining within a sample. The ASVs common to both samples and their taxonomic assignment are summarized as the within-sample percent abundance. The other ASVs column denotes the within-sample percent abundance for ASVs not common to both samples.

b Two ASVs are common to both samples, and thus, the total no. of ASVs postfiltration is less than the sum of ASVs within the interior and surface samples.

### Data availability.

16S rRNA amplicon DNA sequences from this study have been uploaded to the National Center for Biotechnology Information Sequence Read Archive (SRA) under the accession numbers SRX18763179 (interior sample), SRX18763180 (surface sample), and SRX18763181 (glass bead control sample).
